# Short‐term exposure to ethanol induces transcriptional changes in nontumorigenic breast cells

**DOI:** 10.1002/2211-5463.13693

**Published:** 2023-08-18

**Authors:** Georgiana M. Miller, Tyler S. Brant, James A. Goodrich, Jennifer F. Kugel

**Affiliations:** ^1^ Department of Biochemistry University of Colorado Boulder CO USA

**Keywords:** 4sU‐seq, alcohol, breast cancer, transcription

## Abstract

Breast cancer is a leading cause of cancer‐related deaths in women. Many genetic and behavioral risk factors can contribute to the initiation and progression of breast cancer, one being alcohol consumption. Numerous epidemiological studies have established a positive correlation between alcohol consumption and breast cancer; however, the molecular basis for this link remains ill defined. Elucidating ethanol‐induced changes to global transcriptional programming in breast cells is important to ultimately understand how alcohol and breast cancer are connected mechanistically. We investigated induced transcriptional changes in response to a short cellular exposure to moderate levels of alcohol. We treated the nontumorigenic breast cell line MCF10A and the tumorigenic breast cell lines MDA‐MB‐231 and MCF7, with ethanol for 6 h, and then captured the changes to ongoing transcription using 4‐thiouridine metabolic labeling followed by deep sequencing. Only the MCF10A cell line exhibited statistically significant changes in newly transcribed RNA in response to ethanol treatment. Further experiments revealed that some ethanol‐upregulated genes are sensitive to the dose of alcohol treatment, while others are not. Gene Ontology and biochemical pathway analyses revealed that ethanol‐upregulated genes in MCF10A cells are enriched in biological functions that could contribute to cancer development.

## Graphical abstract

Studies have established a link between alcohol consumption and breast cancer; however, the molecular basis for this relationship is poorly understood. Using tumorigenic and nontumorigenic breast cells, we evaluated changes in transcription induced by a short exposure to moderate levels of alcohol. Alcohol‐upregulated genes in nontumorigenic cells are enriched in biological functions that could contribute to cancer development.

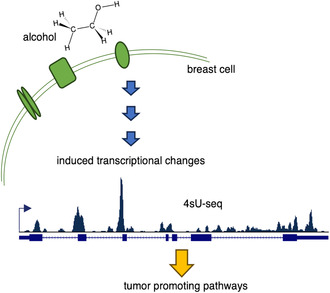

**Abbreviations**
4sU: 4‐thiouridine
B2M: beta‐2‐microglobulin
DLG5: discs large MAGUK scaffold protein 5
DNMT1: DNA methyltransferase 1
EMT: epithelial–mesenchymal transition
EtOH: ethanol
FGF18: fibroblast growth factor 18
GAS1: growth arrest specific 1
GNG11: G protein subunit gamma 11
GO: gene ontology
HIST1H4L: histone H4 gene
KDM2B: lysine demethylase 2B
KEGG: Kyoto Encyclopedia of Genes and Genomes
NDUFA13: NADH:ubiquinone oxidoreductase subunit A13
SPEN: spen family transcriptional repressor
STAT3: signal transducer and activator of transcription 3
STEAP1: six‐transmembrane epithelial antigen of prostate 1
TBX2: T‐box transcription factor 2
TET1: Tet methylcytosine dioxygenase 1
TF: transcription factor
TMEM158: transmembrane protein 158

Breast cancer is the most common cancer for women worldwide and the second most common cancer overall [1]. Like other cancers, breast cancer involves alterations in genome stability, proliferation, apoptosis, motility, and angiogenesis, which together dictate malignant growth [2]. At the molecular level, several genetic abnormalities or ‘driver mutations’ have been found to contribute significantly to breast cancer [3]; however, the picture of breast cancer initiation and progression is far more complex. Studies suggest that thousands of genes may contribute to breast cancer pathophysiologies [3, 4]. Additionally, environmental factors, diet, and chemicals can contribute to the disease, thereby creating a complex interplay of genetics and environment [5, 6, 7]. These relationships are particularly important to understand because they represent modifiable risk factors.

Alcohol is considered a behavioral and dietary risk factor for breast cancer. Epidemiological studies dating back to the 1980s have shown a positive correlation between alcohol consumption and breast cancer [8, 9]. Although differing in their details, methods, and precise conclusions, a positive association between alcohol consumption and breast cancer is widely accepted [8, 10, 11, 12, 13, 14]. Moreover, this association is observed with only moderate alcohol consumption—averaging as little as one drink per day. Despite wide consensus on the link between alcohol consumption and breast cancer, the molecular changes in cells that contribute to this relationship are still ill defined.

Aberrant changes in transcription lie at the heart of many cancerous phenotypes [15] because the accurate transcription of genes is critical for maintaining cellular homeostasis and avoiding irregularities such as rapid proliferation, as seen in cancer cells. Therefore, identifying alcohol‐induced perturbations in the transcription levels of genes critical to cancer progression could advance our molecular understanding of the link between alcohol consumption and breast cancer. Evidence suggests that alcohol will trigger changes to the transcriptional program in breast cancer cells. For example, treating breast cancer cells with alcohol induces transcription of specific estrogen‐responsive genes such as GREB1, PR, and pS2 [16, 17]. Ethanol treatment has also been shown to promote cellular proliferation [17, 18, 19, 20], to stimulate the EGFR, JNK, and p38 MAPK signaling pathways [19, 21, 22], and to increase the expression of genes involved in the epithelial–mesenchymal transition (EMT) [19, 22]. Our goal is to unravel the global transcriptional changes that occur in response to ethanol in both tumorigenic and nontumorigenic breast cells. Currently, genome‐wide studies that provide a comprehensive view of the immediate changes in breast cell transcription in response to ethanol are lacking.

Here, we identified global transcriptional changes in breast cells that occur in response to a few hours of moderate exposure to ethanol. To do so, we leveraged 4sU‐seq (metabolic labeling with 4‐thiouridine (4sU) followed by deep sequencing), which captures changes to ongoing transcription. Three types of human breast cells were treated with ethanol for 6 h: (a) the preneoplastic mammary epithelial cell line MCF10A, (b) the estrogen receptor‐negative epithelial derived breast cancer cell line MDA‐MB‐231, and (c) the estrogen receptor‐positive epithelial derived breast cancer cell line MCF7. Differential transcription analysis showed statistically significant transcriptional changes upon ethanol treatment of MCF10A cells, but not MDA‐MB‐231 or MCF7 cells. Select upregulated genes in MCF10A cells were validated by qRT‐PCR and shown to be responsive to ethanol, some in a dose‐dependent manner. Gene Ontology, pathway, and transcription factor binding site analyses showed that ethanol‐upregulated genes in MCF10A cells participate in biological pathways known to affect cancer progression.

## Materials and methods

### Ethanol treatment and 4sU metabolic labeling of cell lines

MCF10A cells (ATCC, American Type Culture Collection) were cultured in 5% CO_2_ at 37 °C and maintained in DMEM/F12 supplemented with 5% horse serum, 20 ng·mL^−1^ epidermal growth factor, 0.5 μg·mL^−1^ hydrocortisone, 100 ng·mL^−1^ cholera toxin, 10 μg·mL^−1^ insulin, and 1% penicillin–streptomycin. MCF7 cells (ATCC) were cultured in 5% CO_2_ at 37 °C and maintained in DMEM supplemented with 10% FBS and 1% penicillin–streptomycin. MDA‐MB‐231 cells (ATCC) were cultured in 0% CO_2_ at 37 °C and maintained in L15 supplemented with 10% FBS and 1% penicillin–streptomycin.

Prior to ethanol treatment, MCF10A and MDA‐MB‐231 cells were seeded in 10 cm dishes and cultured in antibiotic‐free media for 48 h until cells reached ~ 70–80% confluency. MCF7 cells were seeded in 10 cm dishes and cultured in antibiotic‐free and phenol‐red‐free media for 48 h until cells reached ~ 70–80% confluency. Cells were treated with 0.3% ethanol (EtOH) or left untreated for 6 h. To add ethanol to the media, 70% of the media covering the cells was pipetted into a 15 mL conical and EtOH was added to a final concentration of 0.3%. The remaining 30% of media covering the cells was aspirated and the 0.3% ethanol‐containing media was then immediately added to the cells and incubated for 6 h. At 4 h into the incubation, 200 μM 4sU (final concentration) was added to the cells using a similar approach. Six hours after the EtOH was added, cells were harvested. Total RNA was extracted using TRIzol according to the manufacturer's instructions.

### Biotinylation and purification of 4sU‐labeled RNA

Methods were similar to those previously described [23]. One hundred microgram of total RNA was added to a 250 μL MTS‐biotin labeling reaction with 1× Biotinylation buffer (10 mm HEPES, pH 7.5, 1 mm EDTA), and 5 μg of MTSEA biotin‐XX (Biotium, Freemont, CA, USA) dissolved in 20% DMF. Reactions were nutated at room temperature for 30 min in the dark, and then, phenol/chloroform extracted, followed by ethanol precipitation. The RNA pellets were dissolved in 50 μL of dH_2_O and incubated at 65 °C for 10 min followed by rapid cooling on ice. Biotinylated 4sU‐labeled RNA was separated from unlabeled RNA using the μMacs Streptavidin Kit (Miltenyi, Bergisch Gladbach, Germany). One hundred microliter of μMacs Streptavidin Microbeads was added to the 50 μL of RNA and nutated at room temperature for 15 min. Meanwhile, μMacs μColumns (Miltenyi) were equilibrated twice with 100 μL of Equilibration Buffer for nucleic acid applications (Miltenyi). Samples were applied to the μColumns and flowthroughs were collected. The μColumns were then washed twice with 500 μL of high salt wash buffer (100 mm Tris–HCl, pH 7.4, 10 mm EDTA, 1 m NaCl, and 0.1% Tween‐20). 4sU‐labeled RNA was eluted from the μColumns with 100 μL of freshly prepared 100 mm DTT. A second elution was performed 5 min later with another 100 μL of 100 mm DTT. The eluates were ethanol precipitated, and RNA pellets were resuspended in 20 μL of dH_2_O.

### Illumina sequencing and computational analysis

Sequencing libraries were constructed using the CORALL Total RNA‐Seq Library Prep Kit with RiboCop (Lexogen, Vienna, Austria) and unique dual indexing. Libraries were run on an Illumina NovaSEQ 6000 instrument obtaining 1 × 150‐bp reads. Reads were mapped to the human genome (hg38 assembly) using hisat2 [24], version 2.1.0 in the L, 0, 0.15 alignment mode plus trimming 61 bps from the 3′ ends and 10 bps from the 5′ ends where read quality was low. Meta‐analyses and per‐gene quantification of mapped reads were performed using the suites of tools available in homer [25] and in bedtools (v2.26.0) [26]. Tag directories in homer were created from the sam files generated by hisat2. The command analyzeRepeats.pl in homer was used to determine reads per kilobase for the RefSeq annotation and normalized to 75 million reads, and Pearson correlations were calculated to evaluate the similarity between replicates. To allow visualization of the mapped reads in the ucsc genome browser, bedgraph files were generated using the makeucscfile command in homer. The command featureCounts, run through the galaxy platform [27] using sam files as input, was used to generate raw read count files to use as input for deseq2 analysis [28]. deseq2 analysis, also run through the galaxy platform, was used to generate differential expression data for each treated condition relative to the untreated condition per cell line. The deseq2‐calculated fold changes for the top 10 000 transcribed genes in the untreated condition for each cell line, identified using normalized read counts from deseq2, were used for k‐means clustering with cluster 3.0. The output values were log_2_‐transformed and displayed as heat maps using javatreeview. Gene Ontology analyses were performed using the functional annotation tool in david bioinformatics resources 6.8 [29, 30], with the Gene Ontology category BP_DIRECT and the pathway term KEGG. Enrichment for any given Gene Ontology term or KEGG pathway required a Benjamini–Hochberg corrected *P*‐value < 0.05. The data from david were displayed as bubble plots using srplot (http://www.bioinformatics.com.cn/srplot, an online platform for data visualization). The TF enrichment (Table 1) was performed using shinygo 0.76.3 [31].

**Table 1:** Transcription factors (TF) whose binding sites are enriched in the region 600‐bp upstream of the genes upregulated by ethanol. Analysis was performed with shinygo 0.76.3 [31].

TF	TF family	*P*‐value
CGBP	CxxC	7.6E‐08
DNMT1	CxxC	2.8E‐07
NFYA	CBF/NF‐Y	8.4E‐07
KDM2B	CxxC	3.7E‐06
NFYB	Unknown	5.0E‐06
TET1	CxxC	1.2E‐05
NFYA	CBF/NF‐Y	1.9E‐05
IRF2	IRF	1.4E‐04
E2F1	E2F	2.0E‐04
NRF1	Unknown	4.1E‐04
E2F1	E2F	6.6E‐04
HOXB13	Homeodomain	7.8E‐04
IRF1	IRF	9.6E‐04
E2F2	E2F	9.8E‐04
E2F4	E2F	1.2E‐03
POU2F1	Homeodomain, POU	1.4E‐03
E2F1	E2F	1.7E‐03
MLL	CxxC	2.0E‐03

### qRT‐PCR

MCF10A cells were seeded in 6‐well plates and cultured in antibiotic‐free media for 48 h when cells reached ~ 70–80% confluency. Cells were then treated with 0.1% EtOH, 0.3% EtOH, 0.9% EtOH, or left untreated for 6 h in biological triplicates. Total RNA was extracted from cells using TRIzol (Invitrogen, Waltham, MA, USA). qRT‐PCR reactions were performed in technical duplicate using the Luna Universal One‐Step qRT‐PCR Kit (NEB, Ipswich, MA, USA) and a StepOnePlus Real‐Time PCR system (Applied Biosystems, ThermoFisher, Waltham, MA, USA). The expression level of each mRNA was normalized to B2M (beta‐2‐microglobulin) mRNA levels, and the fold changes in expression between untreated and EtOH‐treated cells were calculated using the ΔΔ*C*_t_ method. Primer sequences are in Fig. S1.

## Results and Discussion

### Short treatment with ethanol induces changes in levels of newly transcribed mRNA in breast cells

**Fig. 1:**
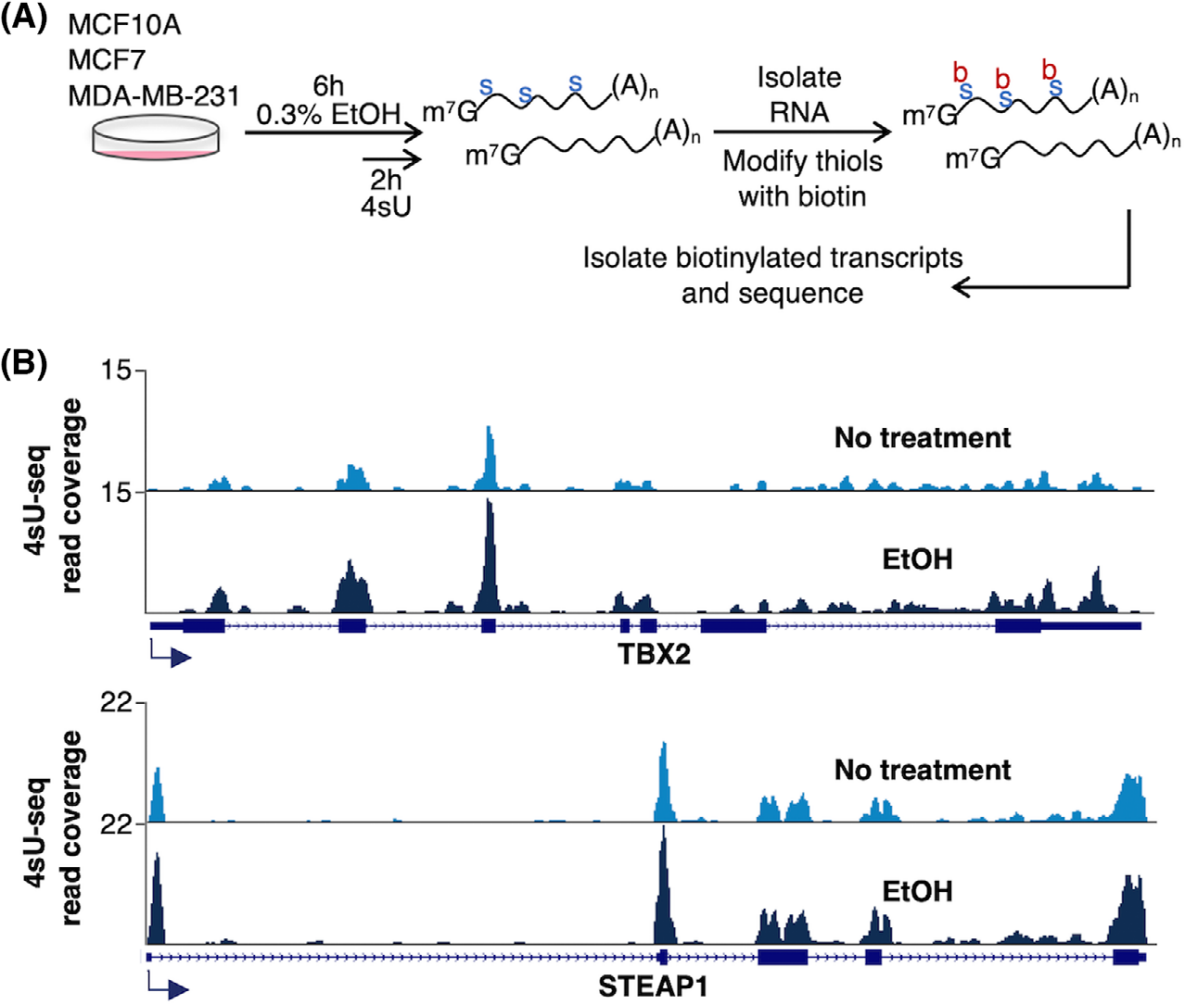
4sU‐seq reveals ethanol (EtOH)‐induced changes in transcription. (A) Schematic of the method shows that EtOH was added to growth media at 0.3% for 6 h, with 4‐thiouridine (4sU) added for the last 2 h of the EtOH treatment. Total RNA was isolated from cells; 4sU‐labeled transcripts (blue s) were biotinylated (red b) and purified using streptavidin beads. RNA libraries were prepared and sequenced in biological triplicate. (B) Representative 4sU‐seq data for the TBX2 and STEAP1 genes in MCF10A cells. Sequencing tracks were generated from the ucsc genome browser display of bedgraph files showing mapped sequencing reads. Within each gene schematic, the rectangles represent exons and arrowheads represent introns.

To determine how ethanol affects early transcription in nontumorigenic and tumorigenic breast cells, we performed 4sU‐seq in three different cell lines: MCF10A, MDA‐MB‐231, and MCF7 (Fig. 1A). 4sU‐seq provides the advantage of capturing changes to new, ongoing transcription during ethanol treatment, whereas total RNA‐Seq captures steady‐state RNA levels and detects changes to both transcription and RNA turnover. All cell lines were treated with ethanol (EtOH) for 6 h, with 4sU added for the last 2 h to enable metabolic labeling of newly transcribed RNA. This approach enabled us to capture early transcriptional responses to alcohol exposure; most prior studies focused on longer time points of ethanol treatment, such as 24 h to days [16, 17, 18, 19, 20, 21, 22, 32]. Ethanol was added to the media at 0.3% (60 mm). This molar concentration of ethanol is greater than the *K*_m_ for alcohol metabolizing enzymes present in breast tissue [33]. Due to the volatility of ethanol and its rapid metabolism in the cell, we quantified the amount of ethanol remaining in the culture media after 6 h using a fluorescent assay and found that the ethanol concentration did not substantially decrease. The 4sU‐labeled RNA from all cellular conditions was harvested in biological triplicate, biotinylated with MTS‐biotin, purified using streptavidin beads, and deep sequenced. The reads were mapped to the human genome, and the replicates sequenced for each cellular condition were similar, as determined by Pearson correlation coefficients (Fig. S2).

**Fig. 2:**
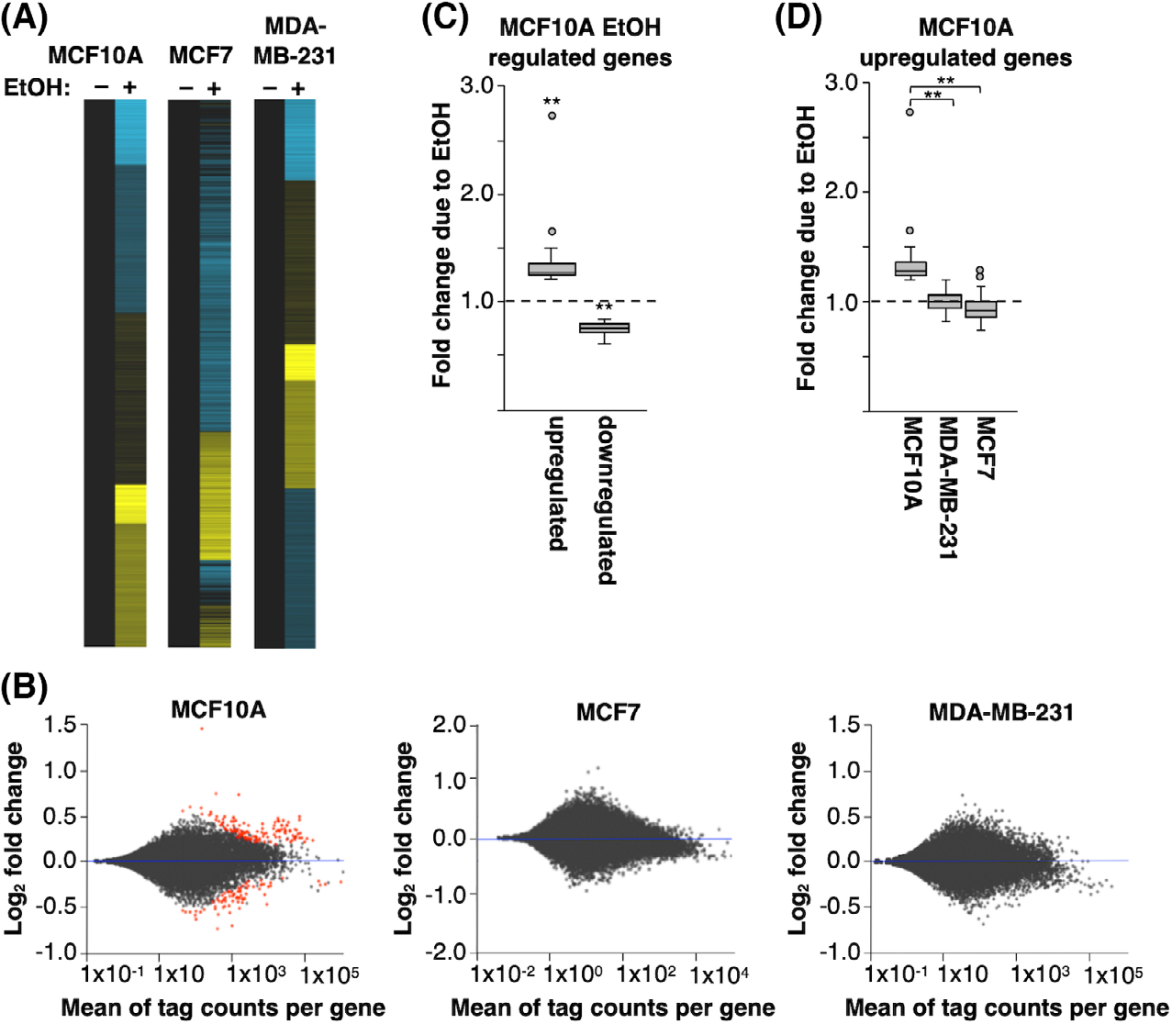
Differential transcription is observed in MCF10A cells treated with EtOH compared with untreated cells. (A) Heat maps show fold changes in transcription after EtOH treatment for the top 10 000 transcribed genes in the untreated condition for each cell type. Genes were k‐means clustered; the untreated condition was normalized to 1.0 (black). Yellow indicates an increase, and blue indicates a decrease. The ranges in fold changes are 0.60–2.73 for MCF10A cells, 0.62–1.65 for MDA‐MB‐231 cells, and 0.58–1.83 for MCF7 cells. (B) Shown are MA plots of RefSeq genes comparing transcript levels in EtOH‐treated versus untreated conditions for each cell line. Each dot represents a gene. Dots highlighted in red are genes with statistically significant differential transcription (Benjamini–Hochberg adjusted *P*‐value < 0.05) in EtOH vs. untreated conditions. (C) Shown is a box and whisker plot depicting, in quartiles, the fold changes for statistically significant (adjusted *P*‐value < 0.05) transcriptionally upregulated and downregulated genes in MCF10A cells after treatment with ethanol. The dashed line represents no change in expression, the circles are outliers, and the ** denotes a *P*‐value of < 0.0001 as compared to 1.0 using a one‐sample two‐tailed *t*‐test. (D) For the list of genes activated in MCF10A cells, the fold change in transcript level after ethanol treatment was plotted for each cell type. The dashed line represents no change in expression, the circles are outliers, and the ** denotes a *P*‐value of < 0.0001 from an unpaired two‐tailed *t*‐test comparing the bracketed samples.

Sequencing tracks of the 4sU‐seq data at two representative genes in MCF10A cells are shown in Fig. 1B. Both TBX2 (T‐Box Transcription Factor 2) and STEAP1 (Six‐Transmembrane Epithelial Antigen of Prostate 1) transcripts show small increases after ethanol treatment. Observing reads within introns is indicative of using 4sU‐seq to capture ongoing transcription. To visualize an overview of the impact of ethanol treatment in each cell line, the fold changes in transcript level for the top 10 000 transcribed genes were plotted in heat maps (Fig. 2A). Both upregulation and downregulation were observed across all three cell lines, with modest fold changes in newly made transcript levels. Observing relatively small fold changes was not surprising given our short windows of ethanol treatment and metabolic labeling with 4sU.

To determine which changes due to ethanol treatment were statistically significant, the deseq2 program was used to perform differential expression analysis [28]. MA plots were generated to quantitatively visualize the range in differential transcription and to highlight statistically significant differences between treated and untreated conditions for each cell line (Fig. 2B). Each gene is a single point, with increases and decreases in transcript levels shown above and below the blue horizontal line, respectively. The statistically significant changes (Benjamini–Hochberg adjusted *P*‐value < 0.05) are highlighted in red. In MCF10A cells, ~ 200 genes showed statistically significant differential transcription after ethanol treatment. Of these, transcripts that were upregulated or downregulated at least 1.2‐fold are listed in Figs S3 and S4, respectively; ranges of the fold changes for these genes are shown in the box and whisker plot in Fig. 2C. A prior study in MCF12A cells (also a nontumorigenic breast cell line) showed that long‐term treatment (1 week) with low ethanol (0.012%) induced up or downregulation of a few hundred transcripts [34], some of which we also observed as differentially regulated after a short ethanol exposure in MCF10A cells.

No ethanol‐induced transcriptional changes were statistically significant in the MDA‐MB‐231 or MCF7 cells. As a point of comparison, for the genes upregulated by alcohol in MCF10A cells, we plotted the fold change due to ethanol treatment in the other two cell types (Fig. 2D). The ethanol‐activated genes in MCF10A cells showed little change in expression due to ethanol treatment in the two other tumorigenic cell types. Our finding that ethanol did not induce statistically significant changes in transcript levels in the two cancerous cell types was arguably surprising given previous literature reports. However, in these prior studies cells were treated with ethanol for 24 h up to weeks, and total RNA was evaluated rather than ongoing transcription [16, 17, 18, 19, 20, 21, 22, 32].

Models to explain the link between alcohol consumption and breast cancer propose that a different cellular response to ethanol occurs in tumorigenic versus nontumorigenic breast cells [33]. Our data suggest this differential response occurs, at least in part, at the level of transcription. One model, known as the cumulative carcinogen model, hypothesizes that alcohol consumption promotes a switch in healthy cells to enter a cancerous‐like state by inducing molecular changes that trigger increased growth, a propensity for metastasis, and apoptosis evasion [2, 33]. A second model, known as the tumor promoter model, hypothesizes that alcohol consumption stimulates molecular changes in preexisting breast cancer cells to promote tumor progression to a clinically diagnosable and invasive state [33]. These models are not mutually exclusive and contributions from both likely explain how alcohol consumption can drive progression of breast cancer. Our data characterize the short‐term differential response to alcohol in cancerous and noncancerous cells.

In evaluating the list of upregulated genes in MCF10A cells (Fig. S3), the enrichment of histone genes is immediately apparent. Indeed, > 30 different histone genes were statistically upregulated by a short treatment of ethanol. The vast majority of histone genes are transcribed during S phase of the cell cycle [35]. Some studies have shown that treating cultured cells with ethanol can lead to changes in the cell cycle [36, 37]. One study with breast cancer cells showed that ethanol treatment caused an accumulation of cells in S phase [18]. This could explain the transcriptional activation of histone genes seen in our 4sU‐seq data. Interestingly, it has been proposed that histone stress, resulting from misregulation of canonical histone genes, can trigger chromosomal instability, which is observed in cancers [38, 39].

### Ethanol increases steady‐state RNA levels of select genes, some in a dose‐responsive manner

From the list of genes upregulated due to ethanol treatment of MCF10A cells, we selected several for further validation, choosing ones with biological functions that could contribute to breast cancer development: TBX2 (T‐Box Transcription Factor 2), TMEM158 (Transmembrane protein 158), DLG5 (Discs Large MAGUK Scaffold Protein 5), and STEAP1 (Six‐Transmembrane Epithelial Antigen of Prostate 1). TBX2 is a transcription factor that regulates cell fate decisions, cell migration, cell cycle progression, and morphogenesis [40, 41]. It is amplified and overexpressed in various human breast cancers and breast cancer cell lines [42, 43, 44]; moreover, ectopic expression of TBX2 in MCF10A cells induces characteristics of the epithelial–mesenchymal transition (EMT) [41]. TMEM158 is an oncogene involved in tumor progression in several cancer types [45, 46]. More recently, TMEM158 was found upregulated in breast cancer patient samples and shown to contribute to EMT and proliferation [47]. DLG5 is important for regulating cell polarity and has been implicated in several cancers. Studies suggest that DLG5 could act both as an oncogene and a tumor suppressor within the same cancer type, making it an intriguing target for further study [48]. STEAP1 is involved in metal ion transport, has a well‐documented role in the early stages of prostate cancer [49] and is overexpressed in human breast cancers [50]. In addition to the aforementioned genes, we also chose to investigate HIST1H4L (Histone H4 gene) as a representative of the 32 histone genes found to be upregulated in our dataset.

**Fig. 3:**
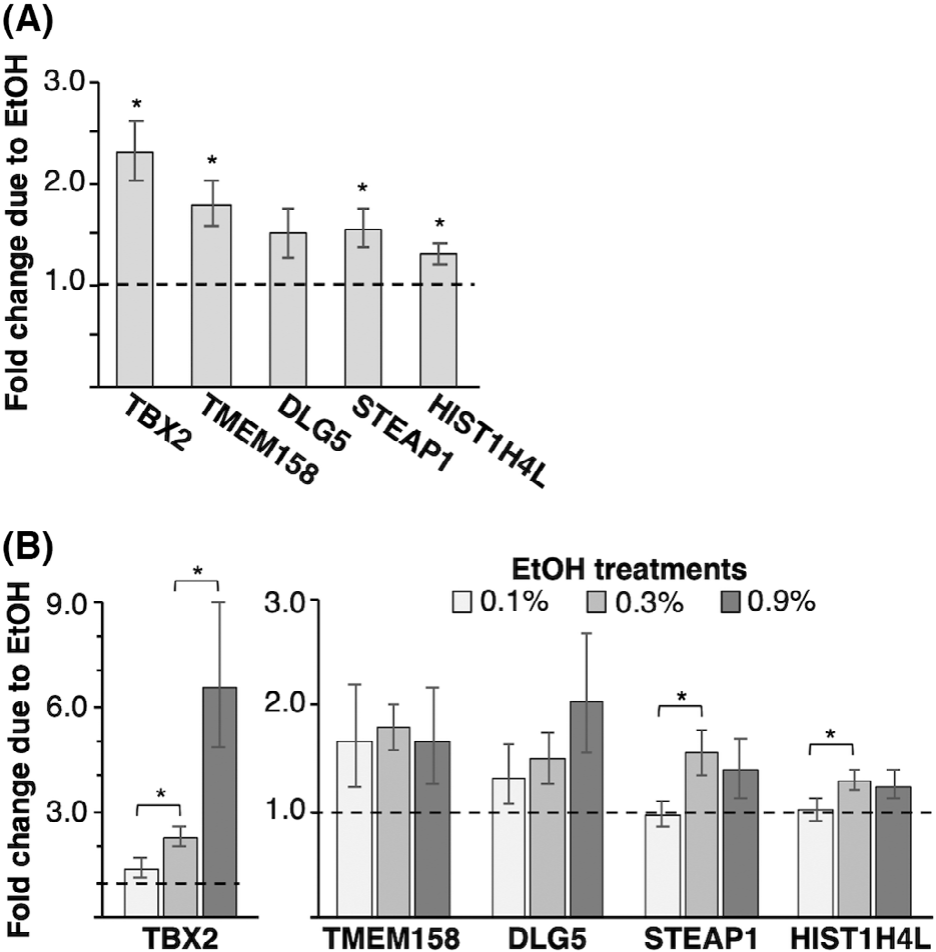
mRNA levels of selected genes show alcohol‐induced upregulation. (A) MCF10A cells were treated with 0.3% EtOH for 6 h, and mRNA levels were determined by qRT‐PCR. Plotted is the average fold change in mRNA, normalized to the control B2M transcript, in EtOH‐treated cells relative to untreated cells. The error bars denote standard deviations (*n* = 3). The dashed line at 1.0 represents no change in mRNA levels. * indicates a *P*‐value < 0.05 compared with 1.0 (no change) determined from a one‐sample two‐tailed *t*‐test. The *P*‐value for DLG5 is 0.06. (B) Upregulation of TBX2 mRNA by ethanol is dose dependent. Average fold change in mRNA expression, normalized to B2M mRNA levels, in EtOH‐treated cells relative to untreated cells is plotted. TBX2 has a different *y*‐axis than the other four transcripts. The error bars denote standard deviations (*n* = 3). * indicates a *P*‐value < 0.05 determined from a two‐sample *t*‐test.

For our selected genes, we tested whether the alcohol‐induced upregulation of transcription could be observed via qRT‐PCR of total RNA harvested from newly treated (0.3% EtOH for 6 h) MCF10A cells compared with untreated cells. As shown in Fig. 3A, all five of the mRNAs increased in abundance after ethanol treatment (i.e., the fold change from untreated to treated was greater than 1.0 (dashed line), although the increase in DLG5 was not statistically significant (*P*‐value of 0.06). Hence, orthogonal assays with different cultures of cells show these genes are upregulated in response to alcohol treatment. We also asked whether upregulation of these genes was dose dependent by comparing changes in transcript levels after treatment with 0.1%, 0.3%, and 0.9% EtOH for 6 h (Fig. 3B). TBX2 showed the strongest dose‐dependent increase in expression. Both STEAP1 and HIST1H4L mRNA levels increased between the 0.1% and 0.3% EtOH treatments, but no additional increase was observed with 0.9% EtOH. TMEM158 and DLG5 did not show dose‐dependent upregulation.

The ethanol‐induced changes in some transcripts were different in magnitude in the 4sU‐seq data compared with the qRT‐PCR data. For example, TBX2 was more strongly upregulated in the qRT‐PCR data, and the opposite was true for DLG5. 4sU‐seq is a more direct assay of ongoing transcriptional changes, whereas qRT‐PCR on total RNA probes steady‐state RNA levels, reflecting the combined effect of transcription and RNA turnover/stability. It is likely that DLG5 is a less stable transcript and TBX2 a more stable transcript, which would explain the difference in the magnitude of ethanol‐induced activation using these two assays. Genes most likely to be identified as differentially upregulated via the 4sU‐seq experiment are those transcribed at a high rate during the labeling period. It is possible that transcriptional upregulation of genes whose RNAs have rapid turnover rates could be dampened or not detected in an experiment that assays total RNA.

### Investigating the biology of differentially transcribed genes in ethanol‐treated MCF10A cells

**Fig. 4:**
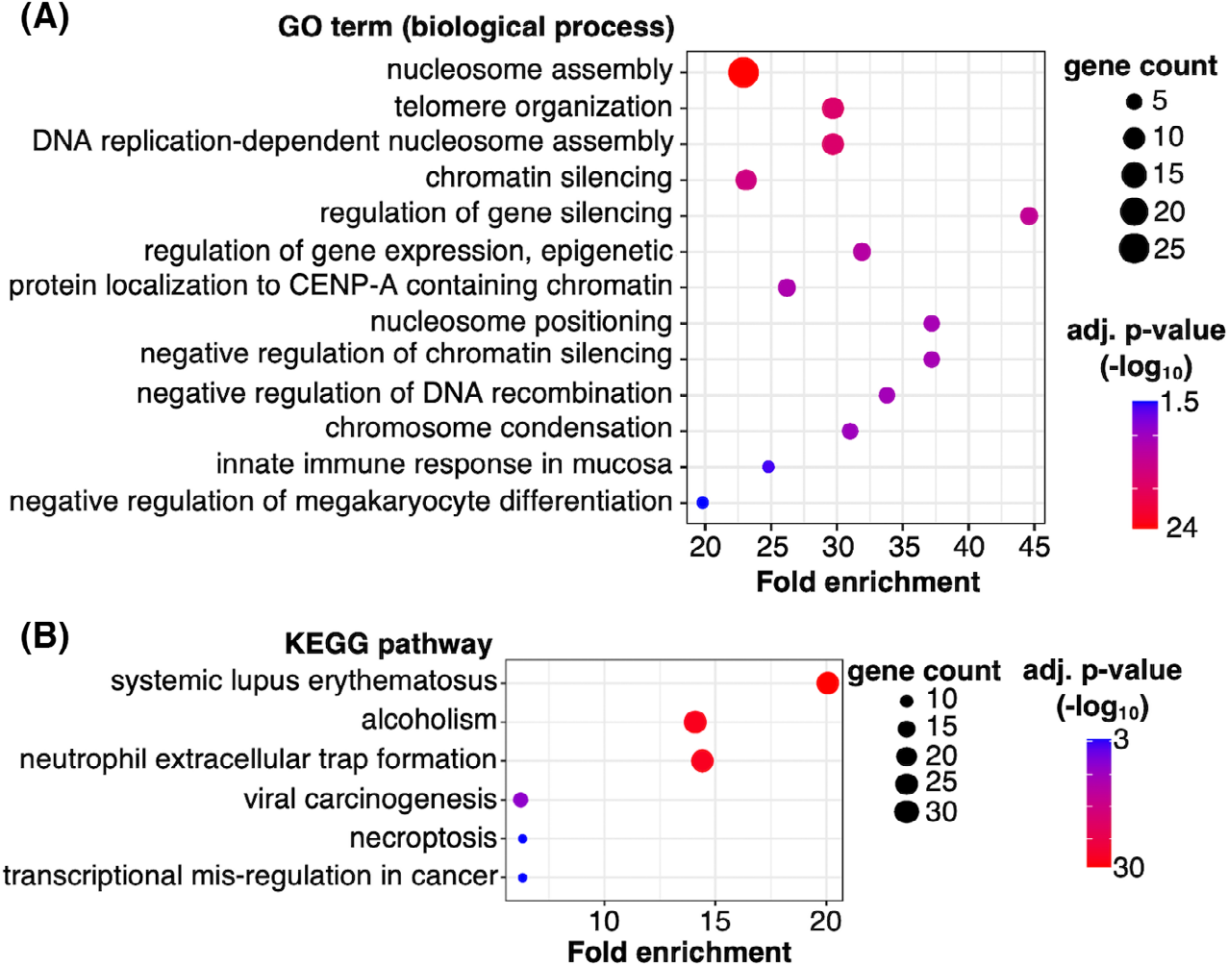
Biological processes and biochemical pathways were enriched in the genes transcriptionally upregulated by ethanol in MCF10A cells. (A) Bubble plot of the enriched biological processes identified by david's Functional Annotation tool. Fold enrichment is the percent of ethanol‐upregulated genes in each category divided by the percent of background genes in each category, where background is the top 10 000 transcribed genes in the dataset. Enriched terms had Benjamini–Hochberg corrected *P*‐values of < 0.05. (B) Bubble plot of the enriched KEGG pathways identified by david.

We next determined whether the differentially transcribed genes in MCF10A cells were enriched in biological functions or cellular pathways known to contribute to cancer initiation and/or progression. We first performed Gene Ontology (GO) analysis for the 100 activated and 62 repressed genes. We identified 13 biological process GO terms that were enriched (Benjamini–Hochberg corrected *P*‐values < 0.05) in the upregulated gene data set; no biological processes were statistically enriched in the downregulated gene data set. Figure 4A shows a bubble plot of the GO terms enriched in the upregulated gene set, where the x‐axis shows the fold enrichment, the size of each point represents the number of genes in each GO term, and the color of each point represents the *P*‐value. The majority of the enriched biological processes in Fig. 4A are nucleosome‐, chromatin‐, epigenetic‐, and gene silencing‐related. This is not surprising since 32 of the upregulated genes encode histone proteins; indeed, most of the statistically enriched GO terms were related to these histone genes.

Considering this, we repeated the GO analysis after removing the 32 histone genes. Using the broadest category list of GO terms, we found that many genes clustered into biological processes directly related to breast cancer tumorigenesis, although they did not have adjusted *P*‐values of < 0.05. These biological processes included cell growth and proliferation (e.g., CCNB2, GAS1, MT1E, NDUFA13, STC1, and TBX2), metal ion transport (e.g., COX17 and STEAP1), cell adhesion (e.g., ATP1B1, CALR, CCNB2, CLDN23, DLG5, HES1, and SERPINE1), tissue morphogenesis (e.g., DLG5, HES1, STC1, and TBX2), cellular response to chemical stimuli (e.g., BAMBI, CALR, GAS1, HES1, MT1E, NDUFA13, OSER1, SERPINE1, and STC1), and negative regulation of signaling (e.g., BAMBI, CALR, CAVIN3, NDUFA13, and SERPINE1). It is possible that transcriptional upregulation of these genes in normal breast cells potentiates tumor development.

Interestingly, some of the upregulated genes are known to exhibit tumor suppressor‐like behaviors. This suggests the early transcriptional response to ethanol in nontumorigenic cells could trigger competing cellular responses, perhaps working to counteract one another. For example, GAS1 (Growth Arrest Specific 1), which promotes growth suppression [51], was upregulated. Also, upregulated was NDUFA13 (NADH:Ubiquinone Oxidoreductase Subunit A13), which keeps the signaling protein STAT3 (Signal Transducer and Activator of Transcription 3) in an inactive state, preventing the potential for oncogenic transformation [52]. Interestingly, this phenomenon of competing effects was also seen for transcriptionally downregulated genes. For example, SPEN (Spen Family Transcriptional Repressor), a known tumor suppressor [53, 54], was downregulated in response to ethanol; however, FGF18 (Fibroblast Growth Factor 18), which promotes cell proliferation, migration, and differentiation [55] was also downregulated. Future mechanistic studies will be required to unravel the functional interplay between tumor‐promoting and tumor‐suppressing genes in response to alcohol treatment.

We also asked whether any of the upregulated genes in MCF10A cells were common to specific cellular pathways. We used KEGG (Kyoto Encyclopedia of Genes and Genomes) Pathway enrichment analysis to identify whether genes were statistically enriched within specific biochemical pathways. Shown in Fig. 4B is a bubble plot of six enriched pathways, most of which have established links to cancer. In addition to transcriptional misregulation in cancer and viral carcinogenesis, both necroptosis (a caspase‐independent mechanism of programmed cell death) and neutrophil extracellular trap formation have been linked to oncogenesis and cancer progression [56, 57]. As with the GO terms, the histone genes dominated the KEGG pathway analysis and were present in all six enriched pathways. It was compelling to find the alcoholism pathway enriched. In addition to the histone genes, GNG11 (G protein subunit gamma 11) is part of the alcoholism KEGG pathway, functioning to mediate signaling in response to acute ethanol exposure [58]. There are links between GNG11 and breast cancer in the literature [59]; whether GNG11 is a molecular link between alcohol metabolism and cancer will be interesting to investigate in the future.

Although we do not have a precise explanation of how increases in mRNA synthesis happen upon ethanol treatment, we considered whether binding sites for transcriptional regulators are present in the DNA sequences upstream of the genes for these mRNAs. We performed an analysis of the transcription factor (TF) binding sites that were enriched upstream of the alcohol‐upregulated genes. Using shinygo software, the region 600‐bp upstream of each of the genes was analyzed for TF binding sites. As shown in Table 1, more than a dozen TF binding sites were enriched, with strong representation from the E2F and CxxC families. TFs in the E2F family bind to the consensus sequence TTTCCCGC [60], while TFs in the CxxC family preferentially interact with unmethylated CpG dinucleotides through a conserved zinc finger‐CXXC domain [61]. The E2F family of transcription factors is best characterized for their functions in cell cycle regulation as part of the CDK4/6‐RB‐E2F pathway that controls the G1/S transition [60]. E2F transcription factors also have an established role in breast cancer, particularly regarding progression, angiogenesis, and metastasis [60, 62], and are being explored as prognostic markers [63]. Links between alcohol and E2F transcription factors have not been established in the literature and warrant future investigation. Many of the CxxC family members in Table 1 are not canonical TFs, but proteins that regulate epigenetics by modifying DNA or histones [e.g., DNMT1 (DNA methyltransferase 1), TET1 (Tet Methylcytosine Dioxygenase 1), and KDM2B (Lysine Demethylase 2B)]. Interestingly, several of these factors have been linked to alcohol‐induced epigenetic changes in a variety of model systems [64, 65, 66]. Studies investigating CpG methylation in breast carcinomas show a strong correlation between decreased DNA methylation and increased alcohol consumption [67]. It is possible the promoter regions of the genes we identified as alcohol‐responsive also have changes to their epigenetic modifications.

Our investigation of alcohol‐induced changes to the cellular transcriptome leads to the question of how ethanol impacts cellular pathways to ultimately control transcription. This remains an unanswered question. In cells, ethanol is primarily metabolized by alcohol dehydrogenases (ADHs) into acetaldehyde, a known carcinogen [68]. Acetaldehyde can form DNA adducts, mitigate antioxidant defenses, and decrease S‐adenosyl methionine availability, the latter negatively impacting histone modification and DNA methylation [68]. Ethanol treatment has also been shown to stimulate the EGFR, JNK, and p38 MAPK signaling pathways in MCF7 and MDA‐MB‐231 breast cancer cells [19, 21, 22]. Alcohol has been shown to interplay with estrogen and/or growth factors and stimulate epigenetic changes [69]. The degree to which each of these mechanisms contributes to ethanol‐induced changes to the transcriptome remains an open area of investigation.

At its core, breast cancer is a disease of abnormal and mutant gene expression. How these abnormalities and mutations contribute to cancer etiology and progression are ongoing questions in the field. Future functional studies of genes we identified as transcriptionally upregulated or downregulated upon ethanol treatment will help mechanistically elucidate how alcohol consumption contributes to breast cancer progression. Combining these mechanistic studies with continued epidemiology studies can help disentangle the relationship between alcohol consumption and an increased risk of breast cancer.

## Conclusions

Together, our data show that short‐term exposure to ethanol induces transcriptional changes in nontumorigenic breast cells. The changes are modest in magnitude and, however, occur in genes with documented roles in cancer development and progression. These studies provide a basis for investigating the molecular relationship between alcohol consumption and increased breast cancer risk.

## Conflict of interest

The authors declare no conflict of interest.

### Peer review

The peer review history for this article is available at https://www.webofscience.com/api/gateway/wos/peer‐review/10.1002/2211‐5463.13693.

## Author contributions

GMM, JAG, and JFK conceived and designed the project. JAG and JFK acquired funding. GMM and TSB performed experiments. GMM, TSB, and JFK analyzed data. GMM and JFK wrote the manuscript. All authors participated in revision and approved the final version.

## Supporting information


**Fig. S1.** Sequences of the qPCR primers written 5′ to 3′.
**Fig. S2.** Pairwise comparison of Pearson coefficients between 4sU‐seq samples.
**Fig. S3.** Transcriptionally upregulated genes in MCF10A cells due to EtOH treatment.
**Fig. S4.** Transcriptionally downregulated genes in MCF10A cells due to EtOH treatment.

## Data Availability

Sequencing data are available in the NCBI Gene Expression Omnibus (https://www.ncbi.nlm.nih.gov/geo/) and are accessible through GEO Series GSE221280.
