# Supplementary material for: Short‐term exposure to ethanol induces transcriptional changes in nontumorigenic breast cells
Source: FEBS Open Bio. 2023 Aug 18;13(10):1941–52. doi: 10.1002/2211-5463.13693 (PMC10549231; doi:10.1002/2211-5463.13693)
Supplement: Supplementary file 1 — Fig. S1. Sequences of the qPCR primers written 5′ to 3′. Fig. S2. Pairwise comparison of Pearson coefficients between 4sU‐seq samples. Fig. S3. Transcriptionally upregulated genes in MCF10A cells due to EtOH treatment. Fig. S4. Transcriptionally downregulated genes in MCF10A cells due to EtOH treatment. [file FEB4-13-1941-s001.pdf]

Supplementary Figures S1-S4

Miller, et. al.
Short term exposure to ethanol induces transcriptional changes in non-tumorigenic breast cells

Gene name	Forward primer	Reverse primer
**B2M**	TGCTGTCTCCATGTTGATGTATC	TCTCTGCTCCCCACCTCTAAG
**TBX2**	CAACATCTCTGACAAGCACGGCTTC	AGGCAGCTTCAGGATGTCGTTG
**TMEM158**	CGAGCCGCTGCATTTCTGCT	CCACCAAGCGTGGACTCAATG
**DLG5**	CTTGTGTGGTGGGAACCTGCAT	CTGCTCAGCAGCTCAGAACTCA
**STEAP1**	CTGTCTTACCCAATGAGGCGATCC	CTCCAAACATCATGCTCAATCCAGGC
**HIST1H4L**	ACATCCAGGGCATCACCAAGCC	ACACTTTAAGAACTCCGCGTGTCTCC

Supplementary Figure S1. Sequences of the qPCR primers written 5’ to 3’

Sample Name	MCF10A 1	MCF10A 2	MCF10A 3
MCF10A 1	1	0.9956	0.9985
MCF10A 2	0.9956	1	0.9956
MCF10A 3	0.9985	0.9956	1

Sample Name	MCF10A_EtOH 1	MCF10A_EtOH 2	MCF10A_EtOH 3
MCF10A_EtOH 1	1	0.9946	0.9939
MCF10A_EtOH 2	0.9946	1	0.9939
MCF10A_EtOH 3	0.9939	0.9939	1

Sample Name	MDA-MB-231 1	MDA-MB-231 2	MDA-MB-231 3
MDA-MB-231 1	1	0.9937	0.9921
MDA-MB-231 2	0.9937	1	0.9941
MDA-MB-231 3	0.9921	0.9941	1

Sample Name	MDA-MB-231_EtOH 1	MDA-MB-231_EtOH 2	MDA-MB-231_EtOH 3
MDA-MB-231_EtOH 1	1	0.9921	0.9859
MDA-MB-231_EtOH 2	0.9921	1	0.9917
MDA-MB-231_EtOH 3	0.9859	0.9917	1

Sample Name	MCF7 1	MCF7 2	MCF7 3
MCF7 1	1	0.9963	0.9955
MCF7 2	0.9963	1	0.9922
MCF7 3	0.9955	0.9922	1

Sample Name	MCF7_EtOH 1	MCF7_EtOH 2	MCF7_EtOH 3
MCF7_EtOH 1	1	0.9970	0.9972
MCF7_EtOH 2	0.9970	1	0.9955
MCF7_EtOH 3	0.9972	0.9955	1

Supplementary Figure S2. Pairwise comparison of Pearson coefficients between 4sU-seq samples show high correlation between biological replicates. Shown are the Pearson correlation coefficients between replicates 1, 2, and 3, calculated by comparing the mapped reads per kilobase for all RefSeq genes, normalized to a constant depth of sequencing for each sample.

Gene Name (protein-coding)	Fold Increase	Gene Name (protein-coding)	Fold Increase	Gene Name (non-coding)	Fold Increase
ADIRF	1.34	HIST1H3J	1.28	ATP1A1-AS1	1.46
ANKRD36C	1.24	HIST1H4B	1.26	LOC100996756	1.66
ATP1B1	1.25	HIST1H4D	1.35	RMRP	1.41
B4GAT1	1.25	HIST1H4L	1.39	RN7SK	1.37
BAMBI	1.28	HIST2H2AC	1.22	SCARNA10	1.32
CALR	1.20	HIST2H3D	1.23	SCARNA2	1.21
CAVIN3	1.41	HIST2H3PS2	1.33	SCARNA5	1.38
CCDC28A	1.43	HIST3H2A	1.31	SCARNA7	1.36
CCNB2	1.21	HIST3H2BB	1.27	SNORA12	1.43
CDCA4	1.21	HIST4H4	1.25	SNORD3A	1.36
CLDN23	1.42	IER5L	1.34	SRGAP2D	1.35
COX17	1.20	ISOC1	1.22	TERC	1.35
DLG5	2.74	KIF4A	1.21		
ELOVL4	1.25	LYPD3	1.31		
FAM102B	1.36	MANF	1.25		
GAS1	1.26	MEIS1	1.34		
GLCE	1.33	MPZL2	1.25		
GNG11	1.25	MT1E	1.32		
H1FX	1.20	NDUFA13	1.31		
HES1	1.22	NELFB	1.22		
HIST1H1A	1.35	OSER1	1.26		
HIST1H1B	1.23	PDE7B	1.50		
HIST1H1C	1.24	RAB20	1.29		
HIST1H1D	1.27	RASL11B	1.23		
HIST1H2AB	1.32	RHOV	1.40		
HIST1H2AC	1.26	RSL24D1	1.20		
HIST1H2AE	1.28	RTN4R	1.37		
HIST1H2AH	1.25	SAP30	1.24		
HIST1H2AJ	1.28	SCARB2	1.25		
HIST1H2AK	1.24	SERPINE1	1.25		
HIST1H2AM	1.25	SLC2A3	1.28		
HIST1H2BB	1.25	SLC2A4RG	1.27		
HIST1H2BE	1.29	SLC45A3	1.31		
HIST1H2BF	1.24	SNAPC1	1.25		
HIST1H2BG	1.21	SNRNP25	1.28		
HIST1H2BH	1.36	SPSB1	1.26		
HIST1H2BI	1.31	STC1	1.39		
HIST1H2BL	1.27	STEAP1	1.40		
HIST1H2BM	1.20	TBX2	1.67		
HIST1H3A	1.34	TMEM158	1.51		
HIST1H3C	1.20	TSC22D1	1.32		
HIST1H3D	1.31	TULP3	1.27		
HIST1H3G	1.27	ULBP3	1.25		
HIST1H3H	1.28	ZNF365	1.46		

Supplementary Figure S3. Transcriptionally upregulated genes in MCF10A cells due to EtOH treatment. Shown are the 100 genes upregulated at least 1.2-fold with a Benjamini-Hochberg adjusted p-value <0.05 after 6 hours of 0.3% EtOH treatment in MCF10A cells identified from DESeq2 analysis after 4sU-seq. The fold-increase in expression in EtOH-treated cells vs. untreated cells for each gene is shown. The column on the right shows non-coding genes.

Gene Name (protein-coding)	Fold Decrease	Gene Name (non-coding)	Fold Decrease
AJUBA	1.32	FBXL19-AS1	1.26
BNIP2	1.50	ILF3-DT	1.26
C16orf72	1.20	LINC00641	1.31
C1orf52	1.36	LINC01943	1.45
CPO	1.44	LOC101928156	1.30
DYRK1A	1.47	LOC101929185	1.28
EFNB2	1.35	LOC105370877	1.34
FGF18	1.63	LOC105373942	1.46
FLVCR1	1.21	LOC105378751	1.47
GLS	1.41	LOC107984243	1.29
HPDL	1.32	LOC107985360	1.30
KLB	1.36	LOC107986148	1.33
LUC7L3	1.41	LOC107986196	1.37
MTHFD2L	1.36	LOC112268030	1.42
NUFIP2	1.35	MGC16275	1.28
NUFIP2	1.21	MINCR	1.23
PRR14L	1.30	MIRLET7A1	1.44
PUM2	1.32	NIPBL-DT	1.20
PURB	1.38	RNA45SN3	1.41
RAB12	1.34	SNHG26	1.27
RABGGTB	1.27	SNORA33	1.26
RFC3	1.48	SNORD22	1.22
RPL11	1.51	SNORD26	1.33
RPL37	1.33	SNORD28	1.36
RPS29	1.29	SNORD29	1.43
RRP15	1.24	SNORD31	1.32
SETD3	1.67	TASOR2	1.28
SHCBP1	1.55		
SON	1.23		
SPEN	1.22		
SPON1	1.36		
TBCA	1.29		
TRIM52	1.23		
TROVE2	1.47		
WDR33	1.25		

Supplementary Figure S4. Transcriptionally downregulated genes in MCF10A cells due to EtOH treatment. Shown are the 62 genes downregulated at least 1.2-fold with a Benjamini-Hochberg adjusted p-value <0.05 after 6 hours of 0.3% EtOH treatment in MCF10A cells identified from DESeq2 analysis after 4sU-seq. The fold-decrease in expression in EtOH treated cells vs. untreated cells for each gene is shown. The column on the right shows non-coding genes.
